# Neuropeptide Y signaling in osteoarthritis pain: neuroimmune mechanisms, joint remodeling, and translational opportunities

**DOI:** 10.3389/fpain.2026.1884062

**Published:** 2026-09-04

**Authors:** Heng Qiu, Li Wang, Danni Luo, Yong Huang

**Affiliations:** 1 Chengdu University of Traditional Chinese Medicine, Chengdu, Sichuan, China; 2 Affiliated Hospital of Chengdu University of Traditional Chinese Medicine, Chengdu, Sichuan, China; 3 The Air Force Hospital of the Western Theater Command of the People’s Liberation Army of China, Chengdu, Sichuan, China

**Keywords:** chronic pain, neuroimmune signaling, neuropeptide Y, osteoarthritis, pain sensitization, subchondral bone, synovitis, translational medicine

## Abstract

Osteoarthritis is a leading cause of chronic musculoskeletal pain, but pain severity is not fully explained by cartilage loss or radiographic damage. Neuropeptide Y is a multifunctional neuropeptide with receptor- and compartment-dependent effects on nociceptive signaling, sympathetic activity, immune regulation, and skeletal remodeling. Direct osteoarthritis-specific evidence links synovial-fluid neuropeptide Y to pain in some human knee osteoarthritis cohorts, NPY2R signaling to chondrocyte hypertrophy and matrix catabolism, and osteocyte-derived neuropeptide Y to experimental osteoarthritis remodeling. By contrast, proposed effects on synovial fibroblast phenotypes, macrophage polarization, central sensitization, and gut-joint communication are supported mainly by mechanistically adjacent non-osteoarthritis models and remain hypothesis-generating. This review synthesizes current evidence with a pain-centered and translational focus, emphasizing receptor specificity, evidence source, and disease context. Neuropeptide Y is best viewed as one candidate node within broader nociceptive, inflammatory, vascular, metabolic, and biomechanical networks rather than as a primary or exclusive driver of osteoarthritis. Translation will require receptor-selective and tissue-selective strategies, local delivery systems, validated multi-marker panels, and phenotype-stratified studies that distinguish pain-related from structure-related effects.

## Introduction

Osteoarthritis is a whole-joint disorder and one of the leading causes of chronic musculoskeletal pain, functional limitation, and loss of mobility in adults. Although cartilage degeneration remains a defining structural feature, clinical severity is shaped by a broader network that includes synovial inflammation, subchondral bone remodeling, altered joint innervation, metabolic dysregulation, and central nervous system amplification of pain. This broader view is especially relevant for pain research because structural damage and pain intensity are frequently discordant: some patients with advanced radiographic disease have modest pain, whereas others experience severe and persistent symptoms despite less extensive cartilage loss (1–12).

Recent epidemiological studies show that osteoarthritis is not only a degenerative joint disease but also a major contributor to years lived with disability and healthcare use. This burden justifies a pain-centered and mechanism-oriented synthesis rather than a cartilage-only discussion (4, 6, 13–16).

Current osteoarthritis management is still dominated by education, exercise, weight control, analgesics, intra-articular therapies, and ultimately joint replacement. These approaches can improve symptoms, but they do not consistently modify the neuroimmune and structural processes that maintain pain or drive progression in defined patient phenotypes. The absence of broadly established disease-modifying therapies has encouraged a search for mediators that connect nociceptive signaling with the local joint microenvironment (14, 15, 17–24). Neuropeptides are attractive in this context because they can function as neurotransmitters or neuromodulators, regulate immune-cell responses, and influence local tissue remodeling (25–28).

Neuropeptide Y is a 36-amino-acid peptide expressed in central and peripheral neurons and in several non-neuronal tissues. Through G-protein-coupled Y receptors, neuropeptide Y participates in appetite regulation, stress responses, sympathetic outflow, immune regulation, vascular biology, and bone metabolism (25, 29–38). The most direct osteoarthritis-specific observations concern human synovial-fluid associations with knee pain, NPY2R-dependent chondrocyte responses, and osteocyte-derived neuropeptide Y in experimental osteoarthritis (26, 28, 39, 40). Evidence for direct neuropeptide Y control of osteoarthritis synovial fibroblasts or macrophages is substantially weaker: relevant studies establish fibroblast and macrophage contributions to painful synovitis, whereas direct neuropeptide Y effects have mainly been demonstrated in non-osteoarthritis immune models (27, 41–44). Accordingly, neuropeptide Y is considered here as a context-dependent candidate signaling node, not as a single linear disease driver.

**Figure 1:**
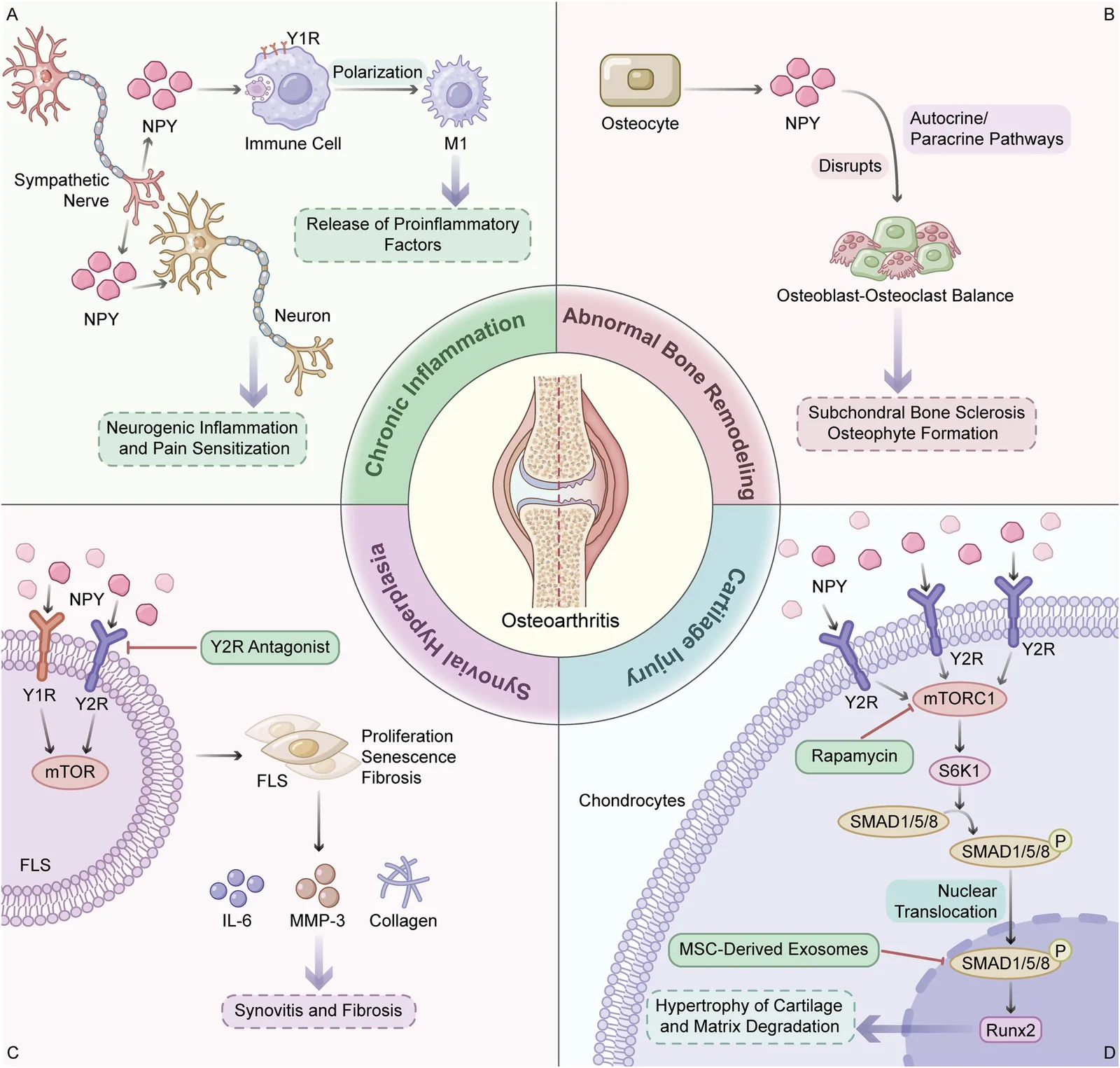
Schematic illustration of the multifaceted roles of neuropeptide Y in osteoarthritis pain and pathophysiology. **(A)** Neuropeptide Y released from sympathetic nerves and neurons may participate in neurogenic inflammation, immune-cell receptor signaling, macrophage polarization, pro-inflammatory mediator release, and pain sensitization. **(B)** Osteocyte-derived neuropeptide Y may act through autocrine/paracrine signaling to disturb osteoblast-osteoclast balance, thereby contributing to subchondral bone sclerosis and osteophyte formation. **(C)** In the synovial compartment, neuropeptide Y-related Y1R/Y2R and mTOR signaling may be associated with fibroblast-like synoviocyte activation, proliferation, senescence/fibrosis, IL-6 and MMP-3 production, collagen deposition, synovitis, and synovial fibrosis; Y2 receptor antagonism is shown as a candidate modulatory strategy. **(D)** In cartilage, neuropeptide Y may activate NPY2R-associated mTORC1/S6K1/SMAD1/5/8/Runx2 signaling, promoting chondrocyte hypertrophy and matrix degradation; rapamycin and mesenchymal stem cell-derived exosomes are shown as candidate intervention nodes. Abbreviations: DRG, dorsal root ganglion; OA, osteoarthritis; NPY, neuropeptide Y; Y1R, neuropeptide Y receptor Y1; Y2R, neuropeptide Y receptor Y2; FLS, fibroblast-like synoviocyte; IL-6, interleukin-6; MMP-3, matrix metalloproteinase-3; mTOR, mammalian target of rapamycin; mTORC1, mammalian target of rapamycin complex 1; S6K1, ribosomal protein S6 kinase beta-1; Runx2, runt-related transcription factor 2.

This review focuses on neuropeptide Y signaling in osteoarthritis pain and whole-joint pathobiology. The central argument is that neuropeptide Y is most clinically informative when interpreted through receptor specificity, cell type, disease phenotype, evidence source, and route of intervention. We therefore synthesize evidence on receptor biology, peripheral and central sensitization, neuroimmune communication, osteocyte-mediated bone remodeling, gut-joint and brain-bone interactions, and translational strategies (Figure 1). The overview of these interconnected mechanisms is summarized in Figure 1.

A pain-centered interpretation also changes how evidence should be weighed. A cartilage-only narrative tends to prioritize pathways that culminate in extracellular matrix degradation, whereas a pain-focused narrative must also ask whether the same pathway alters nociceptor threshold, synovial inflammation, subchondral bone innervation, spinal amplification, or treatment responsiveness. Neuropeptide Y can be studied across several levels of analysis, including synovial fluid and serum biomarkers, immunostaining of nerve fibers, receptor expression in joint tissues, pain-related behavior in animal models, and central nervous system pathways. The manuscript therefore emphasizes mechanistic integration rather than a catalogue of isolated molecular observations. Its main conceptual contribution is to test whether neuropeptide Y can help organize a bridge between nociceptive neurobiology and whole-joint pathology while retaining a clear hierarchy of evidence. This framing is aligned with current pain research priorities, including patient stratification, peripheral-to-central sensitization, neuroimmune communication, and mechanism-based treatment development (8, 9, 45, 46).

## Review scope and interpretive approach

This article is a narrative mechanistic review rather than a systematic review. The aim is not to pool treatment effects or provide a quantitative estimate of efficacy, but to clarify how neuropeptide Y-related pathways may help explain the convergence of pain, inflammation, and structural remodeling in osteoarthritis. Throughout the review, evidence is separated into three levels: (1) direct osteoarthritis-specific evidence from human tissue, clinical cohorts, or osteoarthritis models; (2) osteoarthritis-adjacent mechanistic evidence from joint, bone, or pain studies that does not directly test osteoarthritis; and (3) extrapolated evidence from other inflammatory, metabolic, neuropathic, or postsurgical conditions that is used only to establish biological plausibility. Evidence was interpreted according to tissue compartment, receptor subtype, experimental model, and clinical relevance. Several safeguards were applied: neuropeptide Y biology is pleiotropic; receptor effects may differ by tissue and disease stage; preclinical or associative observations are not treated as clinical proof; and receptor-selective interventions remain experimental in osteoarthritis. The most clinically realistic future applications are therefore likely to involve phenotype selection, local delivery, and biomarker-guided monitoring rather than systemic non-selective modulation.

## Receptor-specific biology of neuropeptide Y in osteoarthritis-relevant tissues

Neuropeptide Y signals mainly through Y-family receptors, among which Y1R and Y2R have received the most attention in osteoarthritis-relevant mechanisms. Y1R is well documented in neuronal, immune, and bone-lineage compartments, but direct receptor-level mapping in human osteoarthritis fibroblast subsets remains insufficient (27, 41, 47). Y2R has direct osteoarthritis-specific support in chondrocytes and osteocyte-related remodeling, and it also has state-dependent functions in sensory neurons in non-osteoarthritis pain models (26, 28, 40, 48). Other receptors, including Y4R and Y5R, may be relevant in metabolic and bone contexts but have not been adequately validated in osteoarthritis. This distribution matters because global stimulation or blockade of the neuropeptide Y system could simultaneously affect pain processing, appetite, vascular tone, immune signaling, and bone turnover (29, 31, 32, 38).

Available data support a receptor-, compartment-, and disease-state-specific model rather than a simple harmful-versus-protective classification. In non-osteoarthritis pain models, Y1R-expressing excitatory dorsal horn interneurons can facilitate neuropathic hypersensitivity, whereas endogenous or exogenous Y1R signaling can also suppress pain under particular circuit conditions (47, 49). Likewise, sensory-neuron Y2R signaling tonically suppresses nociception in uninjured animals but facilitates postsurgical and neuropathic hypersensitivity after injury, demonstrating that receptor function changes with biological state (48). In direct osteoarthritis models, NPY2R signaling promotes cartilage homeostatic disruption, and subsequent work links neuropeptide Y to mTORC1-dependent chondrocyte proliferation and hypertrophy (26, 40). In bone, neuropeptide Y effects depend on whether signaling originates centrally, from sympathetic pathways, or from osteocytes, with osteocyte-derived neuropeptide Y recently implicated in experimental osteoarthritis (28) (Table 1).

**Table 1:** NPY receptor subtypes: osteoarthritis-relevant expression, signaling links, and evidence status.

Receptor subtype	Main osteoarthritis-relevant locations	Representative signaling links	Likely relevance	Evidence status and representative sources
Y1R	Dorsal horn and dorsal root ganglion neurons; immune and bone-lineage cells. Direct mapping in human OA fibroblast subsets remains insufficient.	Gi/o-coupled modulation of neuronal excitability; PKA/Epac and inflammatory signaling.	State-dependent nociceptive modulation; possible immune and skeletal effects.	Direct OA evidence is limited; pain and immune functions are mainly from non-OA models (27, 41, 47).
Y2R	Chondrocytes and osteocyte-related compartments in OA models; sensory neurons in non-OA pain models.	Presynaptic transmitter regulation; mTORC1-linked chondrocyte hypertrophy; osteocyte signaling.	Cartilage catabolism and remodeling; state-dependent nociceptive effects.	Direct OA structural evidence and non-OA sensory evidence (26, 28, 40, 48).
Y4R	Gut and metabolic tissues; OA joint expression is poorly characterized.	Pancreatic-polypeptide and gut-brain signaling.	Possible metabolic or gut-joint relevance.	Indirect, non-OA evidence; no validated OA-specific therapeutic role (31, 86).
Y5R	Central metabolic circuits and bone-related systems; limited OA joint data.	Feeding, energy balance, and neuroendocrine signaling.	Possible systemic metabolic modifier.	Indirect bone/metabolic evidence; OA-specific function remains unclear (29, 38).
Y6R/other	Species-dependent or incompletely characterized.	Insufficiently defined in human OA.	No current translational conclusion.	Evidence-gap row: no adequate OA-specific original source identified.

Citations identify representative original or foundational sources for each row. Rows without direct osteoarthritis evidence are explicitly labeled as indirect or evidence gaps; interpretive synthesis is the authors’ own.

Table 1 summarizes receptor subtype distribution, major signaling pathways, representative original sources, and the level of evidence relevant to osteoarthritis. The main translational implication is that future interventions should be designed around a defined target compartment, such as a pain-dominant sensory phenotype, an inflammatory synovitis phenotype, or a subchondral bone remodeling phenotype, rather than around neuropeptide Y as a single global target.

The Y1R/Y2R distinction is particularly important for mechanistic interpretation. A simplified model might suggest stimulating Y1R for analgesia and blocking Y2R for structural protection, but the available evidence does not support such a universal rule. Y1R-expressing circuits include both endogenous analgesic mechanisms and excitatory interneuron populations associated with pain hypersensitivity, while Y1R on immune cells can alter inflammatory responses outside osteoarthritis (41, 47). Similarly, Y2R can regulate transmitter release in sensory neurons and mediate chondrocyte or osteocyte responses, with direction of effect determined by tissue and injury state (26, 28, 48). Route, dose, timing, and tissue exposure are therefore central determinants of biological effect.

Disease stage is a second receptor-related issue. Early osteoarthritis may involve mechanical stress, synovial activation, and predominantly peripheral sensitization, whereas later disease may include established structural remodeling, central amplification, and persistent disability. Because current neuropeptide Y studies are mostly cross-sectional or preclinical, it is not known whether changes in expression represent a driver, a compensatory response, or a marker of active remodeling. Longitudinal studies should therefore relate receptor expression and neuropeptide Y concentrations to pain duration, synovitis, bone marrow lesions, and structural progression.

## Neuropeptide Y and osteoarthritis pain sensitization

Osteoarthritis pain can arise from variable combinations of peripheral nociceptor activation, local neuroimmune signaling, and central amplification; the relative contribution of each mechanism differs across disease stages and between patients. Neuropeptide Y has the biological capacity to act at several of these levels, but direct osteoarthritis evidence is uneven. In one human knee osteoarthritis cohort, synovial-fluid neuropeptide Y was elevated and associated with pain and radiographic severity, whereas another cohort found no significant relationship between synovial-fluid neuropeptide Y and pain scores (39, 50). These discordant clinical findings argue against a universal neuropeptide Y pain signature and support phenotype-stratified, longitudinal assessment.

Peripheral sensitization is especially relevant in painful osteoarthritis. Synovitis, osteophyte formation, subchondral bone remodeling, and angiogenesis can expose sensory nerve fibers to cytokines, growth factors, prostanoids, altered pH, and mechanical stress. Direct neuropeptide Y mechanisms have been defined mainly outside osteoarthritis: mTOR-neuropeptide Y signaling sensitizes nociceptors after nerve injury, and sensory-neuron Y2R has opposite effects in uninjured versus postsurgical or neuropathic states (48, 51). These studies establish receptor-specific capacity to modify nociception but do not demonstrate that the same mechanism is operative in osteoarthritis joints. Neuropeptide Y should therefore be considered one potential modulator within a larger network that includes nerve growth factor, transient receptor potential channels, inflammatory cytokines, and sympathetic mediators.

Central sensitization adds another layer of complexity. Persistent joint input can alter dorsal horn excitability and descending modulation, leading to pain persistence, spreading pain, and reduced response to peripherally focused treatments. In neuropathic pain models rather than osteoarthritis, Y1R-expressing excitatory dorsal horn interneurons facilitate hypersensitivity, and sensory-neuron Y2R signaling changes after injury (47, 48). These findings provide a defined neural mechanism for hypothesis generation, but direct evidence linking neuropeptide Y to central sensitization in human osteoarthritis is not currently sufficient. Neuropeptide Y is therefore not yet a validated clinical biomarker of central amplification.

Joint innervation and vascular remodeling are additional pain-relevant processes. Human and experimental osteoarthritis studies demonstrate vascular penetration, nerve growth, and osteochondral channels in painful joint compartments (52–55). Recent human knee osteoarthritis tissue profiling further associated worse pain with synovial microvascular dysfunction, altered macrophage states, fibroblast expansion, and neurovascular-remodeling pathways (44). Separately, a non-osteoarthritis study identified macrophage-nociceptor sentinel units around fenestrated synovial capillaries, providing an anatomical basis for neuroimmune surveillance (56). Neither study demonstrates a causal role for neuropeptide Y. Its participation in osteoarthritis neurovascular remodeling should therefore remain a testable hypothesis requiring simultaneous measurement of receptor expression, nerve and vessel density, pain behavior, inflammation, and structural outcomes.

The clinical pain phenotype should be integrated into future neuropeptide Y research. Patients with localized activity-related pain may differ substantially from those with widespread pain, sleep disturbance, anxiety, mechanical allodynia, or poor conditioned pain modulation. These differences reflect variable balances of peripheral, inflammatory, neuropathic-like, and central mechanisms. The inconsistent human synovial-fluid findings also suggest that neuropeptide Y should be assessed alongside quantitative sensory testing, patient-reported pain quality, imaging markers of synovitis, and inflammatory biomarkers rather than interpreted in isolation (39, 50).

In addition, the discordance between structural severity and pain supports the need for mechanism-based phenotyping. Quantitative sensory testing, neuropathic-like pain questionnaires, imaging markers, and inflammatory markers should be combined when evaluating candidate neuropeptide biomarkers (57–60).

Another issue is the relationship between pain and structural change. Neuropeptide Y-related pathways may influence both, but not necessarily in parallel. A treatment that reduces nociceptor excitability might improve pain without slowing cartilage loss, whereas a treatment that limits chondrocyte hypertrophy or subchondral remodeling might not immediately improve established central sensitization. This distinction is essential for trial design. Future studies should define whether the primary endpoint is pain relief, structural modification, synovitis reduction, biomarker change, or a composite phenotype.

## Neuroimmune and synovial mechanisms

The synovium is an important interface between neural signaling and local inflammation in osteoarthritis. Synovial macrophages, fibroblast-like synoviocytes, endothelial cells, and sensory and sympathetic nerve fibers form a heterogeneous microenvironment that can sustain pain and low-grade inflammation. Osteoarthritis-specific studies show that painful synovial regions contain distinct fibroblast subsets with inflammatory, fibrotic, and neuronal-growth programs; fibroblast activation can release mediators that recruit immune cells and sensitize sensory neurons; and worse knee pain is associated with microvascular dysfunction and altered macrophage-fibroblast states (42–44). These studies establish the relevance of the stromal-neuroimmune compartment, but they do not directly show that neuropeptide Y controls it in osteoarthritis. Direct neuropeptide Y actions on immune cells have instead been demonstrated mainly in other inflammatory or metabolic settings (27, 41).

Fibroblast-like synoviocytes can amplify inflammation through interleukin production, matrix metalloproteinase release, extracellular matrix remodeling, and communication with nociceptors. CD44-related pathways are relevant because they connect hyaluronan biology, synovial-cell activation, and joint lubrication (61–63). However, we identified no direct experimental study demonstrating that neuropeptide Y regulates CD44-positive synovial fibroblast phenotypes in osteoarthritis. Accordingly, a neuropeptide Y-CD44-fibroblast relationship is presented only as a proposed, testable mechanistic link. It should be evaluated by receptor mapping and functional perturbation in patient-derived osteoarthritis fibroblast subsets rather than treated as an established pathway.

Macrophages are established contributors to osteoarthritis synovitis, pain, osteophyte formation, and cartilage catabolism (64–66). Those osteoarthritis studies define macrophage biology but do not demonstrate direct regulation by neuropeptide Y. Direct neuropeptide Y evidence comes from non-osteoarthritis systems: immune-cell Y1R signaling modifies inflammatory and metabolic responses in diet-induced obesity, and neuropeptide Y promotes an M2-like program with autophagy and NRF2 activation in human macrophages studied in an atherosclerosis-related context (27, 41). Thus, an NPY-macrophage interaction in osteoarthritis is biologically plausible but remains inferential. It should not be used to support receptor-selective treatment of inflammatory osteoarthritis until direct effects are demonstrated in osteoarthritis macrophages or intact joint models.

Senescence and fibrosis should also be included in the neuroimmune framework. Senescent synovial and chondrocyte populations can release a senescence-associated secretory phenotype that reinforces inflammation and matrix degradation. A recent arthritis-model study reported that rapamycin-responsive neuropeptide Y signaling was associated with senescence and inflammatory pathways, providing a specific experimental link (67). This result has not yet established neuropeptide Y as a driver of senescence in human osteoarthritis. At present, the pathway should be regarded as early, model-dependent evidence that requires validation in human synovium and cartilage (68, 69).

The synovial compartment also provides a practical opportunity for translational sampling. Unlike cartilage, synovial fluid can sometimes be obtained in symptomatic knee osteoarthritis, allowing measurement of neuropeptides, cytokines, and matrix-turnover markers. However, available human studies have produced inconsistent associations between synovial-fluid neuropeptide Y and pain (39, 50). Levels may also be influenced by disease flare, effusion volume, physical activity, sampling timing, and assay performance. Repeated sampling, standardized activity conditions, assay harmonization, and parallel imaging of synovitis are therefore needed before clinical interpretation.

A reciprocal macrophage-nociceptor cycle is biologically credible because neural mediators can alter immune-cell signaling and macrophage-derived mediators can sensitize nociceptors. Recent work has directly demonstrated macrophage-nociceptor organization around synovial capillaries and fibroblast-dependent inflammatory joint pain, but neither study identified neuropeptide Y as the responsible mediator (43, 56). Demonstrating an NPY-dependent cycle in human osteoarthritis will require spatial receptor mapping, ligand-receptor analysis, and functional perturbation in patient-derived synovial cells or organotypic tissue.

## Cartilage and subchondral bone remodeling

The strongest osteoarthritis-specific structural evidence for neuropeptide Y concerns cartilage and osteocyte-related remodeling. In experimental osteoarthritis, neuropeptide Y acts directly through NPY2R to disrupt cartilage homeostasis and accelerate disease, while subsequent work links neuropeptide Y to mTORC1-dependent chondrocyte proliferation and hypertrophic differentiation (26, 40). These studies provide direct receptor-specific evidence and distinguish the cartilage pathway from more speculative synovial mechanisms. Nevertheless, most data remain preclinical, and independent replication in human osteoarthritis cartilage is required before NPY2R can be considered a validated disease-modifying target.

Subchondral bone is not a passive support structure. Osteocytes sense mechanical stress and can regulate bone remodeling, angiogenesis, cartilage metabolism, and pain-related innervation. Direct osteoarthritis evidence was strengthened by a study showing increased osteocyte-derived neuropeptide Y in aging and destabilization-of-the-medial-meniscus models and demonstrating that intermittent fasting relieved osteoarthritis partly by targeting this osteocyte signal (28). Earlier central and skeletal studies establish broader neuropeptide Y regulation of bone metabolism but are not osteoarthritis-specific (29, 70–73). Together, these findings support an osteocyte-neuropeptide Y pathway in experimental osteoarthritis while leaving its relevance to human subchondral pain and structural progression unresolved.

Osteophyte formation and subchondral sclerosis are clinically important because they can alter joint mechanics and may contribute to pain through bone marrow lesions, nerve ingrowth, and local inflammatory signaling. Neuropeptide Y-positive fibers and receptor-related pathways have been described in bone-remodeling contexts, suggesting a possible contribution to osteophyte or sclerosis phenotypes (54, 74–76). However, human evidence linking synovial or serum neuropeptide Y to imaging-defined structural progression remains limited, and the available clinical biomarker findings are inconsistent (39, 50). Future studies should pair neuropeptide Y measurements with magnetic resonance imaging, quantitative pain phenotyping, and longitudinal structural endpoints (Table 2). Table 2 organizes the major cell-type mechanisms and labels direct osteoarthritis evidence separately from extrapolated or hypothesis-generating evidence.

**Table 2:** Cell- and tissue-specific neuropeptide Y mechanisms relevant to osteoarthritis pain and joint remodeling.

Cell/tissue compartment	Neuropeptide Y-related mechanism	Potential OA consequence	Evidence level, sources, and caution
Dorsal root ganglion and spinal circuits	Receptor-specific modulation of nociceptor excitability and dorsal horn processing.	Possible peripheral sensitization or central amplification.	Defined mainly in neuropathic/postsurgical models, not OA (47, 48, 51). Direction depends on receptor and injury state.
Chondrocytes	NPY2R signaling and mTORC1-dependent proliferation/hypertrophy.	Matrix-catabolic activity and structural progression.	Direct experimental OA evidence (26, 40); human validation remains needed.
Fibroblast-like synoviocytes	Proposed interaction with inflammatory, fibrotic, neuronal-growth, and hyaluronan/CD44 programs.	Synovitis, fibrosis, and nociceptor sensitization.	OA fibroblast biology is supported, but direct NPY or NPY-CD44 regulation is not demonstrated (42, 43, 62).
Macrophages	Proposed Y-receptor-dependent modulation of inflammatory or resolution-oriented states.	Synovitis, pain, osteophyte formation, and cartilage catabolism.	OA macrophage roles are direct; NPY regulation is extrapolated from non-OA models (27, 41, 64, 66).
Osteocytes and subchondral bone	Load- and metabolism-related osteocyte NPY signaling.	Subchondral remodeling and possible cartilage-pain coupling.	Direct experimental OA evidence for osteocyte-derived NPY (28), supported by broader skeletal studies (72, 73).
Gut and systemic metabolic axis	Proposed microbiota-neuroendocrine-metabolic coupling.	Systemic inflammation or metabolic-inflammatory OA phenotype.	Hypothesis-generating; direct NPY-microbiota evidence is mainly non-OA (83, 85, 86).

Citations indicate representative source studies. Direct osteoarthritis findings are distinguished from non-osteoarthritis extrapolation and author-generated hypotheses.

A potential strength of the neuropeptide Y framework is its ability to connect mechanical loading with molecular and pain outcomes, especially through chondrocyte and osteocyte pathways. Mechanical stress affects these cells, which communicate with synovium, bone marrow, vascular structures, and sensory nerves. Experimental evidence that osteocyte-derived neuropeptide Y responds to disease-relevant conditions supports one defined link, but it does not show that neuropeptide Y is the principal transducer of mechanical stress (28). Established biomechanical, inflammatory, neurotrophic, and vascular mechanisms remain essential; neuropeptide Y adds one testable neuroendocrine layer to these interacting models.

The cartilage data also need careful interpretation. Chondrocyte hypertrophy is an important feature of osteoarthritis progression, but cartilage tissue is aneural and therefore cannot directly generate pain. The clinical relevance of a cartilage-specific neuropeptide Y pathway depends on its ability to influence other compartments, such as synovium, subchondral bone, osteophytes, or inflammatory mediators that communicate with nerves. Cartilage mechanisms are therefore interpreted together with subchondral bone and synovial mechanisms, rather than as an isolated pathway (54, 77–79).

## Brain-bone and gut-joint axes: systemic amplification of local joint disease

Osteoarthritis pain can be influenced by systemic metabolic and neuroendocrine conditions. Obesity, insulin resistance, aging, stress, sleep disruption, and low-grade inflammation may alter both pain sensitivity and joint-tissue metabolism. Neuropeptide Y is relevant to these conditions because it participates in appetite regulation, sympathetic tone, stress adaptation, and energy balance (32, 80–82). These broad functions do not establish neuropeptide Y as a primary cause of osteoarthritis; they identify one possible modifier within a larger metabolic-inflammatory network.

Brain-bone communication is one example of this systemic framework. Hypothalamic neuropeptide Y circuits regulate sympathetic outflow and bone remodeling in non-osteoarthritis models, whereas skeletal signals may feed back to central pathways (29, 70–72). The osteoarthritis-specific component is narrower: osteocyte-derived neuropeptide Y contributes to experimental osteoarthritis and is modifiable by intermittent fasting (28). A feedback loop among chronic pain, central neuroendocrine signaling, sympathetic activity, and subchondral remodeling remains plausible but has not been directly demonstrated in patients.

**Figure 2:**
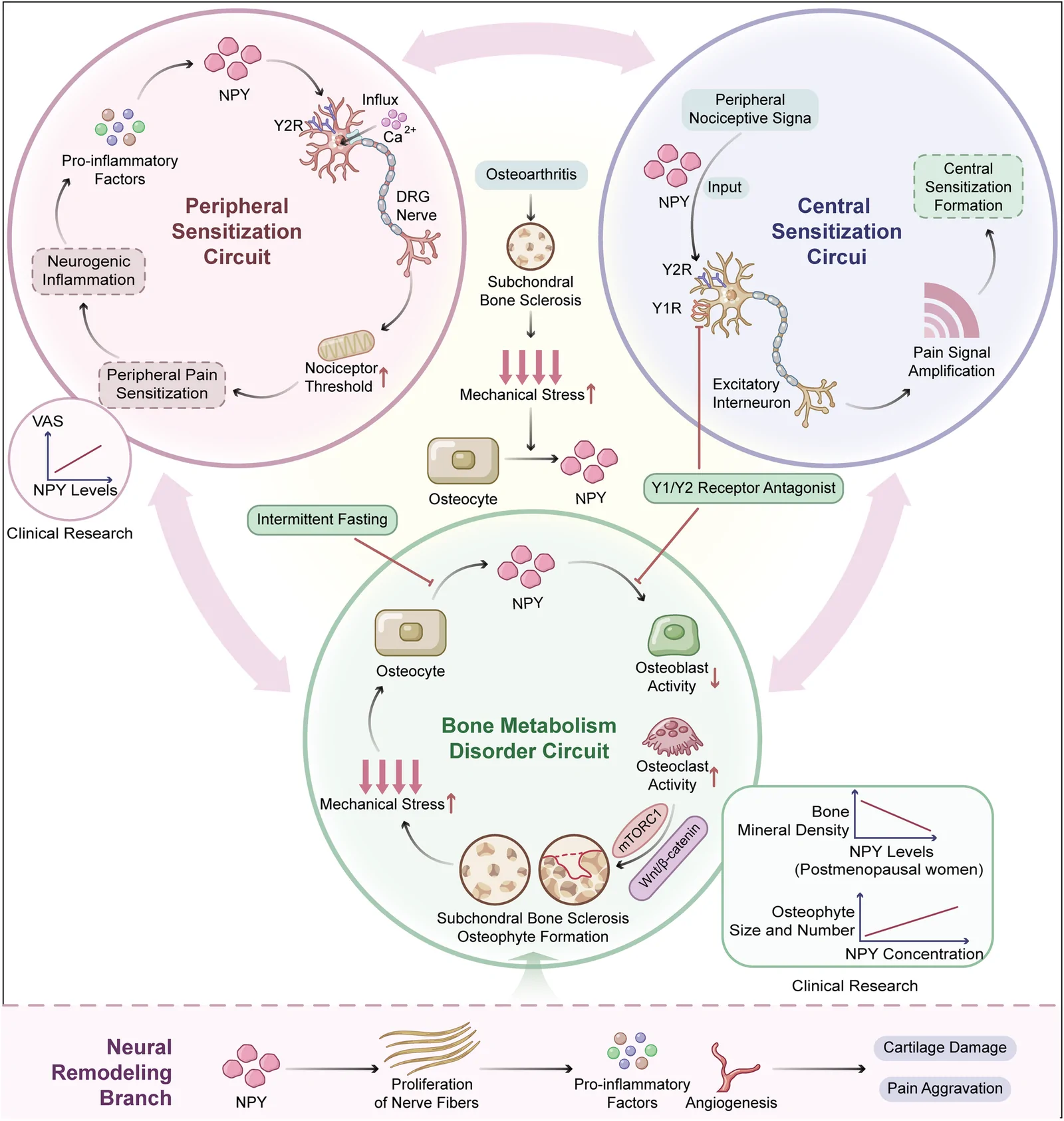
Schematic illustration of neuropeptide Y-mediated neuroendocrine-immune-metabolic-bone-cartilage crosstalk in osteoarthritis. The figure integrates central and peripheral neuropeptide Y signaling with osteocyte activation, synovial inflammation, cartilage catabolism, gut-joint interactions, and candidate therapeutic intervention points. The schematic should be interpreted as a conceptual framework rather than a validated clinical pathway.

The gut-joint axis is another area of interest. Gut microbiota dysbiosis is associated with systemic inflammation, barrier dysfunction, microbial metabolites, and metabolic homeostasis in osteoarthritis-related research (83–85). Direct neuropeptide Y-microbiota evidence derives mainly from other skeletal or metabolic conditions, including experimental postmenopausal osteoporosis, rather than osteoarthritis (86). Consequently, the proposed gut-joint-neuropeptide *Y* axis is hypothesis-generating. Current data do not support neuropeptide Y-directed nutritional therapy for osteoarthritis, although obesity-associated or aging-associated phenotypes may be appropriate populations for future mechanistic studies (Figure 2).

Figure 2 integrates these systemic interactions with the local joint mechanisms described above. The figure should be interpreted as a conceptual framework rather than proof of a single linear pathway. This distinction is important because balanced reviews should identify controversies, gaps, and possible future developments rather than accumulating mechanistic claims without evidence hierarchy.

## Patient modifiers: age and sex

Age is an important modifier of osteoarthritis pain and systemic physiology. Older adults often have multiple contributors to pain, including sarcopenia, altered sleep, metabolic syndrome, osteoporosis, neuropathic features, and central amplification. Because neuropeptide Y participates in stress, appetite, and metabolic regulation, age-related physiology could change its relevance to osteoarthritis. Younger patients with post-traumatic osteoarthritis may instead show a more localized mechanical-inflammatory pattern. These possibilities are hypotheses; age-stratified neuropeptide Y data in osteoarthritis remain insufficient.

Sex-related biology may also modify osteoarthritis prevalence, pain severity, hormonal transitions, and bone remodeling. Neuropeptide Y interacts with metabolic and neuroendocrine systems that may be sensitive to hormonal state, but current evidence does not support sex-specific treatment claims. Future studies should report sex-disaggregated data and test whether neuropeptide Y concentrations or receptor expression differ between females and males, particularly in postmenopausal osteoarthritis and osteoporosis-overlap phenotypes (6, 28, 32).

## Therapeutic implications

Therapeutic interest in neuropeptide Y signaling arises from the possibility of modifying selected pain or remodeling mechanisms, not from evidence that neuropeptide Y is the dominant osteoarthritis pathway. Y1R- and Y2R-directed approaches remain conceptual because receptor effects vary across sensory, immune, cartilage, and skeletal compartments. Direct osteoarthritis evidence currently supports investigation of NPY2R in cartilage and osteocyte-derived neuropeptide Y in experimental remodeling, whereas proposed analgesic or immunomodulatory strategies depend largely on non-osteoarthritis models (26, 28, 40, 47, 48). No receptor-selective neuropeptide Y therapy has established efficacy in human osteoarthritis, and systemic modulation could affect appetite, cardiovascular function, mood, or metabolism.

Local delivery is therefore a key translational requirement. Intra-articular or periarticular delivery could increase joint exposure while reducing central and systemic adverse effects. Nanocarriers, hydrogels, receptor-selective ligands, and RNA-based strategies are possible approaches, but most remain experimental. Mesenchymal stromal cell-derived exosomes and other biologically active vesicles have been studied in osteoarthritis models and may intersect with neuropeptide Y-related pathways, but these approaches require careful characterization, dose control, biodistribution assessment, and safety validation before clinical translation (2, 23, 87–90).

Conventional osteoarthritis treatments should not be interpreted as acting primarily through neuropeptide Y. Exercise, weight management, non-steroidal anti-inflammatory drugs, hyaluronic acid, corticosteroids, and anti-nerve growth factor strategies have established effects through biomechanical, inflammatory, nociceptive, or rehabilitative pathways independent of neuropeptide Y (19, 91–95). Any influence on neuropeptide Y would at present be secondary, indirect, and unproven. Neuroimmune mediators such as nerve growth factor, prostanoids, cytokines, chemokines, ion channels, and other neuropeptides remain equally or more directly implicated in specific osteoarthritis phenotypes. This broader context prevents the review focus from implying that neuropeptide Y is the hidden or exclusive mediator of treatment benefit.

Phenotype selection is likely to determine whether a future neuropeptide Y-directed approach is useful. A pain-dominant phenotype with mechanical allodynia or central amplification may require different targets from an inflammatory synovitis phenotype or a metabolic bone-remodeling phenotype. A practical pathway would combine neuropeptide Y with quantitative sensory testing, imaging of synovitis and subchondral bone lesions, inflammatory and structural biomarkers, and longitudinal pain and function (Table 3). Table 3 summarizes candidate strategies, provides representative source citations, and labels each strategy as direct osteoarthritis evidence, extrapolated mechanism, or conceptual translation.

**Table 3:** Candidate neuropeptide Y-targeted strategies for osteoarthritis: translational rationale and barriers.

Candidate strategy	Primary rationale	Most suitable phenotype hypothesis	Evidence level and representative sources	Major barrier before clinical use
Y2R-directed antagonism	Limit NPY2R-linked chondrocyte hypertrophy and matrix-catabolic signaling.	Structure-progressive OA with cartilage and subchondral remodeling.	Direct preclinical OA cartilage evidence; sensory effects are state-dependent in non-OA pain (26, 40, 48).	Local delivery, off-target neural effects, safety, and human efficacy.
Y1R-directed modulation	Modify selected nociceptive or immune mechanisms.	Pain-dominant or inflammatory phenotype, only after receptor mapping.	Non-OA neuronal and immune evidence (27, 41, 47).	Opposing circuit effects and lack of OA-specific validation.
Local intra-articular delivery	Increase joint exposure while limiting systemic metabolic and cardiovascular effects.	Localized synovial, cartilage, or bone-remodeling phenotype.	Platform-level OA evidence, not NPY-specific (2, 87).	Residence time, biodistribution, formulation stability, and toxicity.
Exosome- or RNA-based modulation	Alter receptor or ligand expression in selected joint cells.	Molecularly defined joint-tissue phenotype.	Early OA platform evidence; direct NPY targeting remains conceptual (87, 89, 90).	Cargo definition, manufacturing, dose control, and off-target effects.
Metabolic, dietary, or microbiota intervention	Modify osteocyte or systemic NPY-related metabolic signals.	Obesity-associated or aging-associated OA.	Experimental OA support for intermittent fasting/osteocyte NPY; microbiota links mainly non-OA (28, 85, 86).	Causal specificity, adherence, and reproducibility.
Combination with established OA care	Use any future NPY-directed intervention as an adjunct to exercise, weight management, or analgesia.	Phenotype-selected multimodal care.	Established therapies have independent mechanisms; NPY interaction is unproven (17, 91, 93).	Demonstrating added benefit beyond standard care.
Biomarker-guided stratification	Combine NPY with pain phenotyping, inflammatory markers, and imaging.	Pain, synovitis, or remodeling endotypes.	Inconsistent human synovial-fluid associations and emerging tissue profiling (39, 44, 50).	Assay harmonization, replication, thresholds, and prospective validation.

Citations identify the evidence supporting each rationale. No neuropeptide Y-targeted strategy has established efficacy in human osteoarthritis; conceptual strategies and author synthesis are explicitly labeled.

Biomarker-guided translation is more realistic than immediate drug development alone. Human synovial-fluid studies illustrate both the opportunity and the limitation: one cohort reported an association between neuropeptide Y, pain, and radiographic severity, whereas another found no association with pain scores (39, 50). A first clinical application, if validated, would therefore be as one component of a multi-marker panel that also includes inflammatory cytokines, cartilage-turnover markers, bone-remodeling markers, quantitative sensory measures, and imaging findings. Neuropeptide Y should not be used as a stand-alone classifier until assays and associations are reproduced across independent cohorts.

Combination therapy should be developed cautiously. Osteoarthritis pain is multifactorial, so a neuropeptide Y-directed intervention is unlikely to replace exercise, weight management, anti-inflammatory therapy, or rehabilitation. Instead, the most plausible future role is adjunctive: a local receptor-selective treatment could be paired with rehabilitation to reduce pain enough to improve function, or with metabolic management in obesity-associated disease. These combinations should be tested in controlled designs because biological plausibility alone is not enough to justify clinical adoption.

## Discussion: translational barriers and future directions

Several barriers must be addressed before neuropeptide Y biology can support clinical intervention in osteoarthritis. First, neuropeptide Y is only one node within interacting networks that include nerve growth factor, prostanoids, cytokines, chemokines, transient receptor potential channels, ion channels, vascular mediators, other neuropeptides, and biomechanical inputs. Second, receptor pleiotropy makes non-selective modulation unlikely to be acceptable because Y receptors influence appetite, anxiety, cardiovascular tone, immune function, and bone metabolism. Third, tissue specificity is substantial: synovium, cartilage, subchondral bone, dorsal root ganglia, and spinal circuits may respond differently to the same ligand. Future studies should therefore use cell-specific and compartment-specific designs rather than whole-joint homogenates or pathway-wide claims.

Clinical phenotyping and evidence hierarchy are additional barriers. Osteoarthritis is heterogeneous, and a neuropeptide Y-related mechanism may be relevant only in subgroups defined by pain phenotype, synovitis, obesity or metabolic dysfunction, bone marrow lesions, age, sex, or disease stage. Direct osteoarthritis evidence is presently concentrated in chondrocyte NPY2R signaling, osteocyte-derived neuropeptide Y in animal models, and inconsistent human synovial-fluid associations (26, 28, 39, 40, 50). Proposed fibroblast, macrophage, spinal, and gut-joint mechanisms rely more heavily on non-osteoarthritis evidence. These distinctions should be preserved until prospective human validation is available.

Future research should prioritize four areas. First, receptor-specific mapping should be performed in human osteoarthritis tissues across disease stages and phenotypes. Second, longitudinal studies should test whether neuropeptide Y levels predict pain progression, structural progression, or treatment response. Third, mechanistic animal studies should measure pain behavior, synovial inflammation, cartilage damage, subchondral bone remodeling, and neural remodeling in the same experimental design. Fourth, early-phase translational studies should assess locally delivered, receptor-selective approaches with careful monitoring for metabolic and neuropsychiatric effects. Together, these steps would move the field from associative biology toward clinically actionable pain mechanisms.

A practical roadmap would integrate human tissue mapping, animal pain behavior, joint imaging, multi-marker biomarker panels, and early safety studies in well-defined clinical phenotypes. This approach would help separate mechanistic plausibility from therapeutic readiness and would reduce the risk of translating receptor biology without patient stratification (96–100).

Evidence quality is also a translational barrier. Claims about global disease burden should be supported by epidemiological or burden-of-disease studies, claims about receptor function should cite primary receptor or disease-model studies whenever possible, and claims about clinical treatment response should cite human clinical trials or well-designed observational studies. This hierarchy is especially important for biomarker proposals involving synovial fluid or serum neuropeptide Y, where reproducibility, assay standardization, and correlation with clinically meaningful outcomes remain unresolved (6, 15, 19, 22).

A final challenge is terminology (101–105). Terms such as neuroimmune axis, gut-joint axis, brain-bone axis, and neuro-osteometabolic axis are useful for conceptual integration, but they can become vague if not anchored to specific cells, receptors, and outcomes. This review therefore uses these terms only after explaining the component pathways, which helps frame the model as a testable mechanistic synthesis rather than speculative terminology (106–110).

## Conclusion

A balanced conclusion is warranted. The review does not argue that neuropeptide Y explains every component of osteoarthritis or that receptor-selective drugs are ready for clinical practice. The clearest osteoarthritis-specific evidence comprises human synovial-fluid observations that are not fully consistent across cohorts, NPY2R-dependent cartilage effects in experimental osteoarthritis, and osteocyte-derived neuropeptide Y in experimental joint remodeling (26, 28, 39, 40, 50). By contrast, direct neuropeptide Y regulation of CD44-positive fibroblasts, osteoarthritis macrophages, human central sensitization, or the osteoarthritis gut-joint axis has not been established. These mechanisms should remain explicitly hypothesis-generating (111–116).

Neuropeptide Y is therefore best understood as a candidate, receptor- and compartment-dependent mediator embedded within broader nociceptive, inflammatory, vascular, metabolic, and biomechanical networks (117–123). Its value lies in organizing testable links between pain and whole-joint remodeling without displacing better-established mechanisms or implying a single-pathway explanation. Translation will require independent human tissue mapping, replicated biomarker studies, receptor-selective perturbation, local delivery, and phenotype-stratified trials that distinguish pain relief from structural modification. Until such evidence is available, neuropeptide Y should be described as a promising mechanistic framework and potential adjunctive target, not as an established disease-modifying treatment (123–125).
